# GIT1 Promotes Axonal Growth in an Inflammatory Environment by Promoting the Phosphorylation of MAP1B

**DOI:** 10.1155/2022/7474177

**Published:** 2022-03-16

**Authors:** Qian Wang, Peng Gao, Hao Liu, Jian Chen, Pengyu Tang, Shujie Zhao, Jin Fan, Yongxin Ren, Guoyong Yin

**Affiliations:** Department of Orthopedics, The First Affiliated Hospital of Nanjing Medical University, Nanjing, China

## Abstract

Spinal cord injury (SCI) is a severe traumatic condition. The loss of the bundle of axons involved in motor conduction in the spinal cord after SCI is the main cause of motor function injury. Presently, axon regeneration in the spinal cord has been studied extensively, but it remains unclear how axon growth is regulated in an inflammatory environment at the cellular level. In the present study, GIT1 knockout (KO) mouse neurons were cultured in a microfluidic device to simulate the growth of axons in an inflammatory environment. The molecular regulation of axon growth in an inflammatory environment by GIT1 was then investigated. We found that the axon growth of GIT1 KO mouse neurons was restricted in an inflammatory environment. Further investigations revealed that in both axons and cell bodies in the inflammatory environment, GIT1 phosphorylated ERK, promoted the entry of Nrf2 into the nucleus, and promoted the transcription of MAP1B, thereby increasing the levels of MAP1B and p-MAP1B and promoting axon growth. We also found that MAP1B could be translated locally in axons and transported in cell bodies and axons. In conclusion, we found that GIT1 regulated axon growth in an inflammatory environment. This provided a theoretical basis for axon regeneration in an inflammatory environment after SCI to develop new treatment options for axon regeneration.

## 1. Introduction

Spinal cord injury (SCI) is a serious traumatic condition with a high mortality and disability rate. Following the advancement of modern construction and transportation technologies, the incidence rate of SCI has increased. According to statistics, in 2016, there were approximately 6–10 million patients with SCIs worldwide. Statistics from the United States (US) suggest that there are approximately 2.5 million cases of SCI in the US alone [1, 2]. The treatment of patients with SCI is expensive, and many patients need long-term care, which causes serious economic burden to families and society. Among the many clinical manifestations of SCI, the loss of motor function is the most serious injury to patients [3, 4]. Macroscopically, a nerve conduction bundle injury of the spinal cord is the cause of motor function loss. Microscopically, axons of motor neurons located in the brain cannot recover and reconnect after axonal injury [5, 6]. Therefore, the key to the treatment of SCI is the promotion of axon regeneration.

After SCI, the regeneration of axons in nerve bundles is affected by external and internal factors. Following SCI, the secretion of Nogo and chondroitin sulfate proteoglycans (CSPGs) at the injured site inhibits axon regeneration [7, 8]. Brain-derived neurotrophic factor (BDNF) and glial cell line-derived neurotrophic factor (GDNF) are often used as foreign factors to promote axon growth in SCI [9, 10]. Axon growth has an internal regulatory mechanism. Some experiments have shown that axon regeneration after SCI can be promoted by regulating the PTEN/mTOR pathway [11, 12]. SOCS3 knockout can promote the germination of corticospinal axons, and codeletion of SOCS3 with PTEN can further promote the germination of axons [13]. Additional studies have found that the effector S6K1 of mTOR plays a role in promoting axon regeneration, while 4E-BP plays a negative regulatory role [14]. An SCI area is located in an inflammatory environment. Previous studies have shown that the levels of a variety of inflammatory factors increase in the cerebrospinal fluid after SCI [15]. In zebrafish with successful spinal cord regeneration, TNF-*α* and IL-1*β* regulate the regeneration of spinal cord axons [16]. However, the regulatory mechanism of axon growth in an inflammatory environment has not been studied in depth.

At present, in the research on the extracellular environment of neurons at the cell level, the cell body and axon are present in the same culture environment, which cannot simulate an axon-only inflammatory environment after SCI. In recent years, the use of microfluidic cell culture devices in the research on nerve cells has gradually increased [17]. This device can physically separate the cell body from the axon end, which is more conducive to the study of axons. Our previous studies showed that the GIT1 protein played a protective role during SCI [18]. We also demonstrated that GIT1 could control the inflammatory response by regulating ERK/NRF2 in bone regeneration [19]. Previous studies have reported that the GIT1 protein played an important role in axon development [20]. However, the effect of the GIT1 protein on axon growth in an inflammatory environment remains to be studied in depth. The present study was aimed at determining how axon growth is regulated by the GIT1 protein in an inflammatory environment at the cellular level.

## 2. Methods

### 2.1. Animal and Animal Model

The GIT1^−/−^ (C57BL/6 background) mice used in the experiment were obtained from the University of Rochester, NY, USA (Aab Cardiovascular Research Institute, Department of Medicine). GIT1 wild-type (WT) littermates were used as a control. All animal experiments and procedures were approved by the Animal Committee of the First Affiliated Hospital of Nanjing Medical University. The contusion model of SCI has been described in our earlier work [21]. Eight-week-old male GIT1 knockout (KO) and WT mice (*n* = 4) were used. The mice were anesthetized by isoflurane inhalation and underwent laminectomy at segment T8. A 5 g rod with a spinal cord impactor to stabilize the spine was dropped from a height of 6.5 cm, and the exposed dorsal surface of the spinal cord sustained heavy fall injury (68097, RWD, USA). After impact, the successful induction of SCI was verified by body shaking and tail, hind limb, and body swinging. Following SCI, the bladder function of the mice was impaired. Micturition was assisted twice a day until bladder function recovered.

### 2.2. Behavioral Assessment

#### 2.2.1. Footprint Analysis

Gait and motor coordination were evaluated 28 days after the operation. The front and rear claws were painted with different colors. The mice were then placed in a narrow passage filled with a white paper and encouraged to walk in a straight line. The walking patterns obtained were photographed with a digital camera, and representative images were used for motor coordination evaluation.

#### 2.2.2. Basso Mouse Scale Behavioral Analysis

The recovery of hind limb motor function was evaluated by a Basso Mouse Scale (BMS) method of measuring hind limb joint activity, trunk position and stability, front and rear limb coordination, foot placement, toe space, and tail position. The mice were placed in an open field, and two researchers, blinded to the treatment, recorded the activities of the mice. The scores were calculated preoperatively and at 7, 14, 21, and 28 days after SCI.

#### 2.2.3. Swimming Test

A swimming test was used to evaluate the recovery of motor function after SCI. The researchers placed the mice in a water tank filled with water and trained them to swim from side to side. Forelimb dependence, hind limb movement and alternation, body angle, and trunk stability were recorded and evaluated according to the Louisville Swimming Scale. Each mouse was tested twice, and the average score was taken as the final score.

### 2.3. Immunofluorescence

The cultured cells were fixed with 4% paraformaldehyde (PFA) for 20 min. After incubation with Triton X-100 in PBS solution (0.3%, *w*/*v*) for 10 min for breaking the cell membrane, the cells were blocked by incubating with goat serum PBS solution (10%, *v*/*v*) at room temperature for 30 min, followed by overnight incubation with primary antibodies at 4°C. Subsequently, the samples were incubated with secondary antibodies at room temperature in the dark for 1 h. After DAPI staining, the cells were observed by a fluorescence microscope, and images were captured. Quantitative analysis was performed using ImageJ software. Frozen tissue sections were fixed with PFA after rewarming. The remaining steps were the same as those used for cellular immunofluorescence assay. In each experiment, we measured the fluorescence intensity of 200 cell bodies and 100 axons to determine the protein expression under different treatments. Each experiment was repeated four times independently. In the microfluidic device, to quantitatively analyze the protein expression of cell body and axon, the fluorescence intensities of approximately 200 cell bodies and approximately 200 axons were measured at the soma side and axon side, respectively. The experiment was repeated at least 4 times.

### 2.4. IL-1*β* and TNF-*α* Level Determination

After SCI modeling in 6-week-old male GIT1 WT and KO mice, the cerebrospinal fluid was extracted before modeling and at 2, 4, 6, and 24 h after modeling. The level of mouse IL-1*β* and TNF-*α* proteins in each cerebrospinal fluid sample was determined using mouse IL-1*β* and TNF-*α* ELISA kits (ab197742 and ab208348, respectively; Abcam, Cambridge, UK) according to the manufacturer's instructions.

### 2.5. Neuron Culture and Lentivirus Transfection

Primary cortical neurons were extracted using a previously described procedure [22]. Embryonic mice were taken on day E18. The cerebral cortex was dissected, and the meninges were stripped and digested with trypsin. The neurons were then inoculated in Petri dishes coated with polylysine. After 4 h, serum-free 96% nerve medium containing B27, glutamine (0.5 mM, Thermo Fisher Scientific), penicillin (100 IU/mL), and streptomycin (100 mg/mL, Thermo Fisher Scientific) was replaced. To separate axons from neurons, a microfluidic device (SND450, Xona Microfluidics) was used [17]. The microfluidic device was placed on the Petri dish coated with polylysine in advance, and neurons were inoculated at the density of 6 × 10^5^ neurons per well. The other steps were the same as mentioned earlier. The medium was changed every alternate day. The concentrations of IL-1*α* and TNF-*β* used to culture neurons in an inflammatory environment were 50 ng/mL and 200 ng/mL, respectively. Lentivirus infection was used for the process of overexpression or knockdown in neurons. The amount of virus was calculated according to the optimal multiplicity of infection (MOI), and the serum-free medium mixed with virus and polybrene was incubated with cells for 6 h. The medium was then changed to normal medium, and the follow-up experiment was performed 48 h later.

### 2.6. Measurement of Axon Length

We defined an axon as a neurite that was at least three times longer than any other neurite. We used the Fiji software to measure the distance from the soma to the center of the growth cone. The data are expressed as mean ± SEM of the percentage of the control group (∼400 neurons). For neurons cultured in microfluidic devices, we defined the length of axons as the distance from the edge of the microgroove to the growth cone. At least 200 axons were measured in each culture, and the experiment was repeated five times. The final results are expressed as mean ± SEM of the percentage of the control group.

### 2.7. Western Blotting

Briefly, the samples were separated on a 4–12% gradient Bis-Tris SDS-PAGE gel and transferred to a polyvinylidene difluoride membrane (SEQ00010; EMD Millipore, Burlington, MA, USA). The membrane was then blocked with bovine serum albumin (5%, *v*/*v*), followed by incubation with the primary antibodies at 4°C overnight. The primary antibodies used were ERK (1 : 1,000, Cell Signaling Technology [CST]), p-ERK (1 : 1,000, CST), GAPDH (1 : 1,000, Abcam), Nrf2 (1 : 100, Abcam), H3 (1 : 1,000, CST), MAP1B (1 : 1,000, Thermo Fisher Scientific), and p-MAP1B (1 : 1,000, Thermo Fisher Scientific). The blots were then incubated with an appropriate secondary antibody at room temperature for 2 h (Thermo Fisher Scientific). Immunoreactive bands were observed with an enhanced chemiluminescence (ECL) reagent (Thermo Fisher Scientific), and the density of protein bands was semiquantified with the ImageJ software.

### 2.8. PCR

The total RNA was extracted from different samples. Each sample was reverse transcribed with 1 *μ*g of total RNA by using a reverse transcription kit, and the total DNA was diluted 1 : 4 in water. The cDNA was amplified on a 7900 real-time PCR system (Applied Biosystems, Foster City, CA, USA) by using 2 *μ*L diluted cDNA and iQ™ SYBR® Green Supermix (Bio-Rad, Hercules, CA, USA). Each sample had three replicates. The target genes were normalized with GAPDH as the standard, and the relative expression level was determined according to the normalized CT value.

### 2.9. Chromatin Immunoprecipitation

After cell treatment, 1% formaldehyde was used to crosslink proteins and DNA. The cells were incubated in a lysis buffer (150 mM NaCl, 25 mM Tris pH 7.5, 1% Triton X-100, 0.1% SDS, and 0.5% deoxycholate), and protease inhibitors were added. The lysate was reduced to 500–1,000 bp by ultrasound, and Nrf2 antibodies were added and immunoprecipitated at 4°C overnight. After immunoprecipitation, the DNA was heated at 65°C overnight to reverse formaldehyde crosslinking. Finally, an MAP1B promoter-specific primer sequence was used for RT-PCR (forward: 5′-ACCAAGTGAGTGAGCAGAGC-3′ and reverse: 5′-CAAGCAACAACAGCAGTGGC-3′).

### 2.10. Statistical Analysis

Statistical analysis was performed using the GraphPad Prism 8 (GraphPad Software Inc., San Diego, CA, USA) and SPSS v23 (SPSS, Chicago, IL, USA). Data analysis was conducted using the GraphPad Prism 8, and all data are expressed as mean ± SEM. Statistical evaluation between the two groups was performed using unpaired Student's *t*-test. The factorial design ANOVA was used to study the interaction between GIT1 and the inflammatory environment during axon growth. For all tests, *P* < 0.05 was considered to be statistically significant. Two-tailed *P* values were used for all the tests.

## 3. Results

### 3.1. Recovery of Neurological Function after SCI in the GIT1 KO Group Was Worse than That in the WT Group

**Figure 1:**
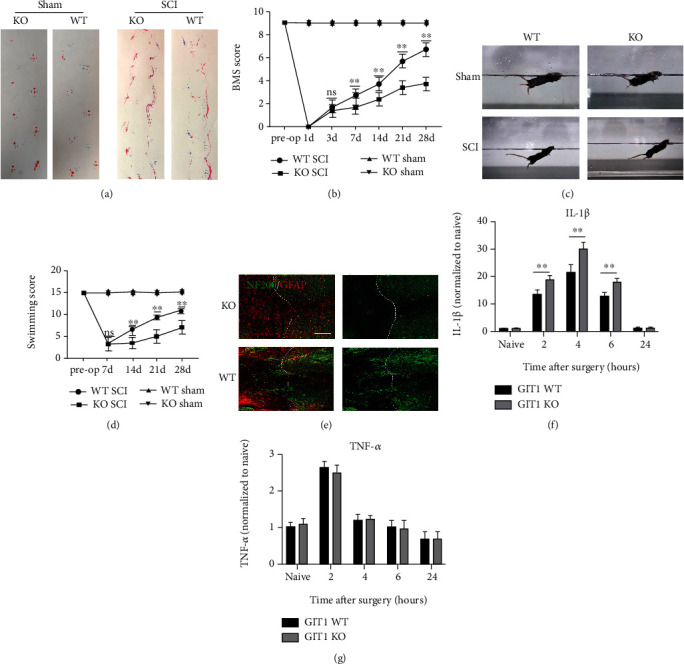
The recovery of neurological function after spinal cord injury in the GIT1 knockout (KO) group was worse than that in the wild-type (WT) group. (a) Representative footprints of mice at day 28 postinjury. (b) The Basso Mouse Scale (BMS) was used to functionally grade mice in different groups up to 28 days postinjury. (c, d) The Louisville Swimming Scale (LSS) score was used to functionally grade mice up to 28 days postinjury. (e) Representative coimmunostaining of NF200 and GFAP in the injured spinal cord at day 14 postinjury (scale bar = 200 *μ*m). (e, f) IL-1*β* and TNF-*α* levels in the cerebrospinal fluid at different time points postinjury.

To study the effect of GIT1 on neural function recovery after SCI, we analyzed the gait after the induction of SCI in mice. After SCI, the functional recovery of the lower limbs in GIT1 KO mice was worse than that in the WT group (Figure 1(a)). We also measured the BMS score of different groups. The BMS score showed that the motor function of the GIT1 KO group was poor (Figure 1(b)). We further used the water maze test to analyze neural function and found that the recovery of neural function in the GIT1 KO group was worse than that in the WT group (Figures 1(c) and 1(d)). To study the effect of GIT1 on the nerve bundle in SCI, we used a spinal cord strike model in mice. The nerve bundles were fluorescently stained with NF200 at different time points. On day 14, we found that axon regeneration in the injured area in the KO group was less than that in the WT group (Figure 1(e)). We measured the biochemical parameters of the cerebrospinal fluid after SCI. ELISA showed that the levels of inflammatory factors in the cerebrospinal fluid were increased significantly in the WT and KO groups after SCI. The IL-1*β* levels in the KO group were higher than those in the WT group, but there were no differences in TNF-*α* levels between the two groups; this finding was similar to that reported in previous studies [22] (Figures 1(f) and 1(g)). Therefore, GIT1 had a protective effect on the recovery of neural function after SCI.

### 3.2. GIT1 Protects Axon Growth in the Inflammatory State

**Figure 2:**
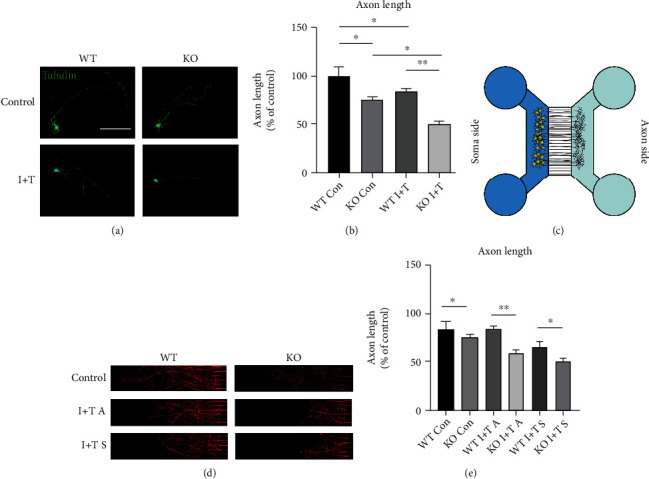
GIT1 protects axon growth in the inflammatory state. (a) Immunofluorescence assay shows the intact axon in different groups (scale bar = 100 *μ*m). (b) Quantitative analysis of axon length in different groups. (c) Mode diagram of the microfluidic device. (d) Immunofluorescence assay of axon ends of cultured neurons with microfluidic devices in different groups (scale bar = 500 *μ*m). (e) Quantitative analysis of axons of cultured neurons with microfluidic devices. ^∗^*P* < 0.05, ^∗∗^*P* < 0.01.

After SCI, the nerve bundle was damaged, and the nerve axon was found to be necrotic in an inflammatory environment. We simulated the growth of axons in an inflammatory environment in vitro. We observed that after the same number of days in a normal culture environment, the axon length of the GIT1 KO group was shorter than that of the WT group. This has been proven in previous studies [20]. When inflammatory factors (IL-1*β*+TNF-*α*) were added to the culture medium, the axons of the KO and WT groups were shorter than those in the normal culture environment (Figures 2(a) and 2(b)). The factorial design ANOVA showed that GIT1 interacts with the effect of inflammatory environment on axon growth. This indicated that GIT1 had a unique protective mechanism against axon growth in an inflammatory environment as compared to that in a normal culture environment. To study the growth of axons in an inflammatory environment, we used microfluidic devices (Figure 2(c)) to physically separate cell bodies and axons. IL-1*β* and TNF-*α* were added at the cell body end and axon end, respectively, to observe the effect on the axons. In the WT group, the growth of axons was inhibited by the addition of inflammatory factors at the soma side and axon side (Figure 2(d)). Compared to the WT group, the growth of axons in the KO group was significantly inhibited (Figure 2(e)). Similar to the previous observation, the factorial design ANOVA showed that GIT1 interacted with the effect of inflammatory environment on axon growth. Thus, GIT1 had a unique protective mechanism against axon growth in an inflammatory environment.

### 3.3. GIT1 Promotes Nrf2 Entry into the Nucleus by Activating ERK in an Inflammatory Environment

**Figure 3:**
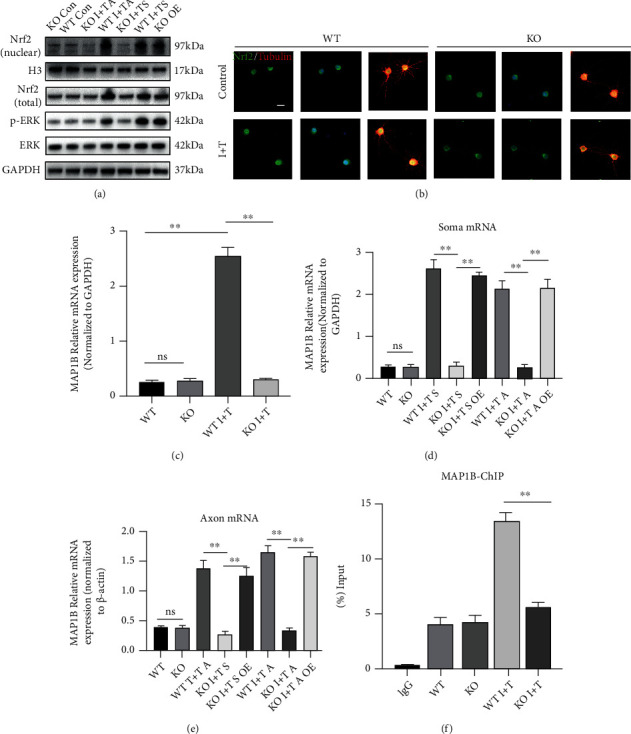
GIT1 promotes Nrf2 entry into the nucleus and promotes MAP1B mRNA transcription. (a) Expression of p-ERK and Nrf2 (nuclear and total) proteins in different groups was determined by western blotting analysis. (b) Immunofluorescence assay showed Nrf2 entry into the nucleus of neurons in different groups (scale bar = 20 *μ*m). (c) The mRNA level of MAP1B in neurons cultured in ordinary Petri dishes in different groups. (d, e) The mRNA level of MAP1B in the soma and axons of neurons cultured in a microfluidic device in different groups. (f) Recruitment of Nrf2 to the MAP1B promoter was assessed by a chromatin immunoprecipitation assay with the indicated antibodies. ^∗^*P* < 0.05, ^∗∗^*P* < 0.01.

Previous studies have shown that in macrophages, GIT1 promoted the phosphorylation of ERK in an inflammatory environment, thereby promoting the nucleation of Nrf2 [19]. Previous results thus suggest that GIT1 has a protective effect on axon growth in an inflammatory environment. Therefore, we detected its protective mechanism in cortical neurons. In a normal environment, there was no difference in ERK phosphorylation between the KO and WT groups. However, in an inflammatory environment, ERK phosphorylation increased, but compared to that in the WT group, it was decreased in the KO group. ERK can be phosphorylated by adding inflammatory factors either at the cell body end or at the axon end. Overexpression of GIT1 was also found to prevent the decrease in ERK phosphorylation in the KO group (Figure 3(a), line 4). Previous studies have shown that ERK activation could promote Nrf2 entry into the nucleus; therefore, we detected the level of Nrf2 entry into the nucleus. In the inflammatory environment, Nrf2 entry into the nucleus was detected by adding inflammatory factors at the cell body end or at the axon end in the WT group, but Nrf2 did not enter the nucleus in the KO group (Figure 3(a), line 1). Cellular immunofluorescence assay also revealed that GIT1 promoted the entry of Nrf2 into the nucleus in the inflammatory state (Figure 3(b)). Therefore, we speculate that GIT1 regulated axon growth mainly through a signaling pathway mechanism by promoting the entry of Nrf2 into the nucleus in an inflammatory environment.

### 3.4. GIT1 Promotes MAP1B mRNA Transcription in an Inflammatory Environment

The MAP1B protein can promote axon growth [23]. Nrf2, as a transcription factor of MAP1B, can promote its transcription [24]. Therefore, we examined the effect of GIT1 on MAP1B expression. There was no significant difference between the WT and KO groups in neurons cultured in ordinary Petri dishes under a normal environment. In the inflammatory environment, the expression of MAP1B mRNA was significantly increased in the WT group but not in the KO group (Figure 3(c)). We continued to add inflammatory factors at the cell body end and axon end and used a microfluidic device to detect the expression level of MAP1B mRNA at the cell body end and axon end. We found that when inflammatory factors were added at either end, the expression level of MAP1B mRNA at the cell body end increased significantly in the WT group, but there was no change in the KO group; moreover, MAP1B mRNA continued to increase after the overexpression of GIT1 (Figure 3(d)). Similarly, we detected MAP1B mRNA at the axon end and obtained the same results (Figure 3(e)). Through chromatin immunoprecipitation (ChIP), we found that the binding of Nrf2 to the MAP1B promoter region increased in the inflammatory environment as compared to the normal environment but not in the KO group (Figure 3(f)).

### 3.5. GIT1 Promotes the Expression of the MAP1B Protein in an Inflammatory Environment

**Figure 4:**
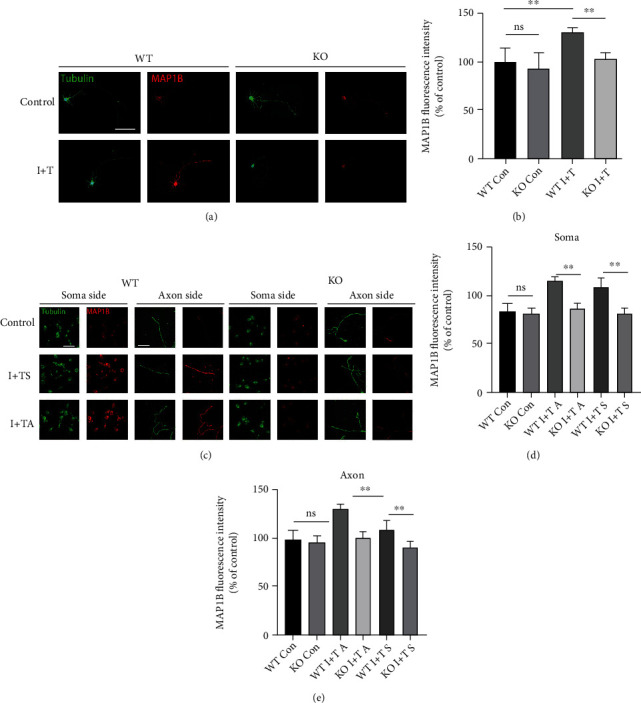
GIT1 promotes the expression of the MAP1B protein in the inflammatory environment. (a, b) Immunofluorescence assay and quantification of MAP1B in neurons cultured in ordinary Petri dishes in different groups (scale bar = 100 *μ*m). (c–e) Immunofluorescence assay and quantification of MAP1B in the soma and axon ends of cultured neurons with microfluidic devices in different groups (scale bar = 50 *μ*m). ^∗^*P* < 0.05, ^∗∗^*P* < 0.01.

We further detected the expression level of the MAP1B protein by immunofluorescence assay. Neurons were cultured in ordinary Petri dishes. The expression level of MAP1B in neurons in the inflammatory environment increased when compared with that in the normal culture environment, but the expression level of MAP1B in the KO group did not increase (Figures 4(a) and 4(b)). By using microfluidic technology, we added inflammatory factors at the cell body end and axon end and observed the expression of MAP1B in the cell body and axon by immunofluorescence assay. The results suggested that the expression of the MAP1B protein in the cell body and axon could be promoted by adding inflammatory factors at the cell body end and axon end. In the GIT1 KO group, the addition of inflammatory factors at the cell body end or axon end did not increase MAP1B expression (Figures 4(c)–4(e)). This showed that even if there were only axons in the inflammatory environment, they could still cause a cell response and promote the expression of MAP1B in the cell body and axon.

### 3.6. MAP1B Phosphorylation Decreased in the GIT1 KO Group under an Inflammatory Environment

**Figure 5:**
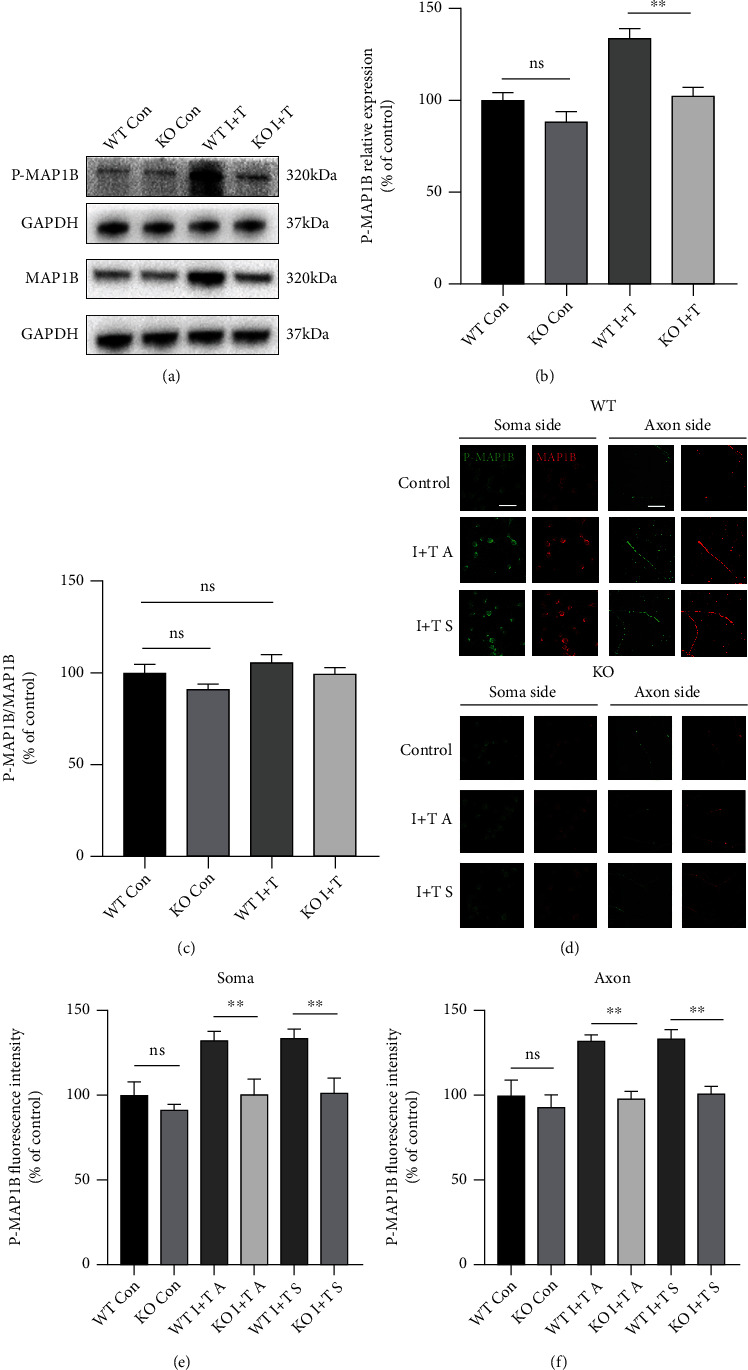
MAP1B phosphorylation decreased in the GIT1 KO group under an inflammatory environment. (a) Expression of MAP1B and p-MAP1B proteins in different groups was determined by western blotting analysis. (b) Quantitative analysis of p-MAP1B in different groups. (c) The ratio of p-MAP1B to total MAP1B. (d–f) Immunofluorescence assay and quantification of p-MAP1B in the soma and axon ends of cultured neurons with microfluidic devices in different groups (scale bar = 50 *μ*m). ^∗^*P* < 0.05, ^∗∗^*P* < 0.01.

MAP1B mainly promotes axon growth through phosphorylation [25]. Therefore, we investigated the phosphorylation level of MAP1B. First, we used western blot analysis to detect the phosphorylation of MAP1B in neurons cultured in ordinary Petri dishes. We observed that the phosphorylation of MAP1B in neurons in the inflammatory environment was higher than that in the normal culture, while the phosphorylation in the KO group did not increase in the inflammatory environment (Figures 5(a) and 5(b)). However, there was no significant change in the ratio of phosphorylated MAP1B to the total MAP1B (Figure 5(c)). Therefore, it was preliminarily concluded that GIT1 did not regulate the phosphorylation process, and the increase in phosphorylated MAP1B was mainly due to the increase in the total amount of MAP1B. We further isolated the cell body and axon by microfluidic culture, added inflammatory factors at the cell body and axon ends, and detected the phosphorylation of MAP1B at the cell body and axon ends. We observed that the phosphorylation of MAP1B at the cell body end increased regardless of the addition of inflammatory factors at the cell body end or at the axon end. Similarly, MAP1B phosphorylation at the axon end increased regardless of the addition of inflammatory factors at the cell body end or at the axon end (Figures 5(d)–5(f)). Therefore, we concluded that while GIT1 promoted the expression of the MAP1B protein in an inflammatory environment, phosphorylated MAP1B also increased, which then played a role in promoting axon growth.

### 3.7. MAP1B Can Be Expressed Locally in Axons and Transported through Axons

**Figure 6:**
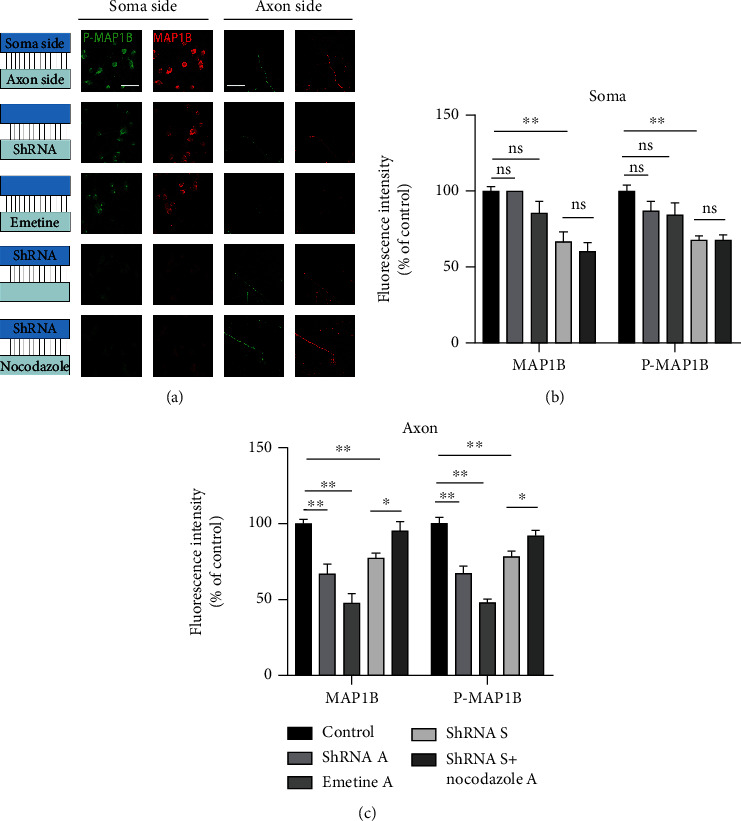
MAP1B can be expressed locally in axons and transported through axons. (a) Immunofluorescence assay of MAP1B and p-MAP1B in the soma and axon ends of cultured neurons with microfluidic devices in different treatments (scale bar = 50 *μ*m). (b, c) Quantification of MAP1B and p-MAP1B in the soma and axon ends of cultured neurons with microfluidic devices in different treatments. ^∗^*P* < 0.05, ^∗∗^*P* < 0.01.

Many studies have shown that several proteins can be translated locally in axons [26]. Therefore, we determined whether MAP1B was locally expressed in axons. We used shRNA to block MAP1B mRNA. If MAP1B mRNA is translated at the axon end, the expression of MAP1B should change after shRNA is added at the axon end. We performed different treatments at both ends to study the expression of MAP1B. First, we added MAP1B shRNA at the axon end and found that the expression of MAP1B at the cell body end did not change significantly. The expression of MAP1B at the axon end, however, decreased significantly (Figure 6(a), line 2). We then added the protein translation inhibitor emetine A at the axon end and again found that the expression of MAP1B at the axon end decreased, which further verified the expression of MAP1B at the axon end (Figure 6(a), line 3). When shRNA was added to the cell body end, the expression of MAP1B at the cell body end decreased significantly, and the expression of MAP1B at the axon end also decreased (Figure 6(a), line 4). We speculated that the decrease at the axon end was because MAP1B was transported to the cell body through the axon after its decrease at the cell body end. Therefore, in our further experiment, after adding shRNA at the cell body end and nocodazole, an axon transport inhibitor, at the axon end, we found that the amount of the MAP1B protein at the axon end was restored (Figure 6(a), line 5). Therefore, our hypothesis that MAP1B could be translated locally in axons and transported through axons was verified.

### 3.8. Overexpression of GIT1 Can Rescue the Negative Effect of GIT1 KO

**Figure 7:**
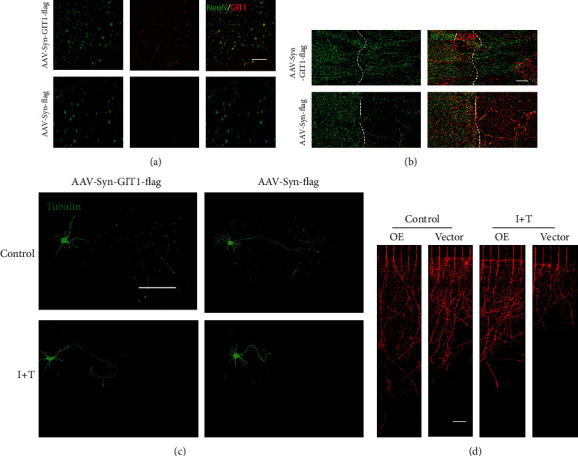
Overexpression of GIT1 can rescue the negative effect of GIT1 KO. (a) Representative image of immunofluorescence assay to detect the GIT1 expression level of KO mice injected with OE virus (scale bar = 100 *μ*m). (b) Representative coimmunostaining of NF200 and GFAP in the injured spinal cord at day 14 postinjury (scale bar = 200 *μ*m). (c) Immunofluorescence assay shows the intact axon in different groups (scale bar = 100 *μ*m). (d) Immunofluorescence assay of axon ends of cultured neurons with microfluidic devices in different groups (scale bar = 500 *μ*m).

To study whether overexpression of GIT1 could rescue the negative effects of GIT1 KO in mice, we injected GIT1 lentivirus into GIT1 KO mice. Three weeks after injection, GIT1 expression was significantly increased (Figure 7(a)), followed by SCI modeling. We observed that axon regeneration was significantly increased in the SCI area injected with GIT1-overexpressing lentivirus as compared to that in the control group (Figure 7(b)). We also studied whether overexpression of GIT1 could rescue the inhibitory effect of GIT1 KO on axon growth in vitro. Our results showed that compared to axons of the control group, the axons of the GITI OE group increased. When IL-1*β*+TNF-*α* (I+T) were added to the culture medium, the axons of both groups became shorter. Moreover, the difference in axon length of the control and I+T in the GIT1 OE group was smaller than that in the control group, indicating that GIT1 rescued neuron growth in the KO group slowly in an inflammatory environment (Figure 7(c)). We also used the microfluidic device to quantitatively analyze axons and obtained the same results (Figure 7(d)). Finally, we concluded that overexpression of GIT1 can rescue the negative effect in GIT1 KO mice and neurons.

## 4. Discussion

The GIT1 protein is a member of the GIT family. As a scaffold protein, GIT1 contains multiple functional regions [27, 28]. The GIT1 protein can regulate the level of mitochondrial autophagy during neuronal ischemia-reperfusion by regulating the phosphorylation of Beclin-1 to protect neurons during spinal cord ischemia-reperfusion [29]. Previous studies have shown that the GIT1 protein can activate Nrf2 of lipopolysaccharide- (LPS-) treated macrophages by regulating the phosphorylation of ERK, thereby reducing the expression of IL-1*β* [19]. Therefore, GIT1 plays an important role in regulating inflammation. Nrf2 is the main transcriptional regulator that regulates the expression of antioxidant- and anti-inflammatory-related genes [30]. Our study showed that GIT1 could promote axon growth in an inflammatory environment and promote the recovery of spinal cord function. We also observed that when axons were in an inflammatory environment, GIT1 promoted the entry of Nrf2 into the nucleus by phosphorylating ERK, thus promoting axon growth. We thus found that GIT1 played an important role in regulating inflammation.

At present, there are different views on the role of IL-1*β* in axons. Studies have shown that IL-1*β* could synergistically promote the growth of axons with NT-3 in vitro [31]. In the IL-1*β* KO mice, the ability of axon regeneration after SCI was enhanced as compared to that in the normal mice [32]. Similarly, there are different views on the role of TNF-*α* in axons. In cultured sensory neurons, TNF-*α* was found to promote axon growth through the NF-*κ*B pathway [33]. Another study reported that the inhibition of TNF-*α* by drugs could promote axonal regeneration in the injured sciatic nerve [34]. The contrasting result may be due to the use of different experimental models, where some were in vivo models and some were in vitro models. In some of the studies, the expression of inflammatory factors in mice was knocked out by gene knockout, while in other studies, the inflammatory factors were investigated together with other factors. An interaction between IL-1*β* and TNF-*α* was also noted [16]. In the present experiment, the inflammatory environment was simulated by adding IL-1*β* and TNF-*α* in vitro. It was found that the axon growth was inhibited by IL-1*β* and TNF-*α* addition.

MAP1B plays an important regulatory role in the growth of axons [23]. Previous studies have suggested that Nrf2, as a promoter of the MAP1B gene, regulated the expression of MAP1B in a Parkinson's disease (PD) model, which then regulated the binding process of microtubules and affects the formation of axons [24]. MAP1B phosphorylation is regulated by a variety of mechanisms. For example, the JNK pathway can regulate MAP1B phosphorylation to stabilize axons [35]. The activation of GSK3 can also regulate the phosphorylation of MAP1B, stabilize axons, and promote axon growth [25]. Our study found a different mechanism involved in the regulation of MAP1B. GIT1 promoted the entry of Nrf2 into the nucleus by regulating the phosphorylation of ERK, thus promoting the transcription and expression of MAP1B. Although the level of phosphorylated MAP1B increased, the phosphorylation ratio did not increase. Therefore, GIT1 increased the levels of phosphorylated MAP1B mainly by promoting the expression of MAP1B. Finally, it promoted the growth of axons.

Studies have shown that the mRNA in the cell body can be transported to the axon by binding with the mRNA-binding protein (mRBP), and the most classic mRBP is the Zipcode binding protein 1 (ZBP-1) [36]. It can then be translated locally in the axon to play a special physiological or pathological role [26, 37]. Axonal injury leads to calcium influx, which initiates the translation of mRNA in axons to repair damage [38]. Previous studies have shown that the MAP1B protein is expressed in axons [24]. However, it remains unclear whether MAP1B is translated locally in axons or transported from cell bodies to axons. In our study, we used a microfluidic device to study the subcellular localization of MAP1B. MAP1B mRNA was detected in axons, and it was found to be translated locally. It was further verified that the MAP1B protein was transported between the cell body and axon under different stimuli. This showed that axons could not only transmit environmental signals to the cell body but also elicit corresponding responses to environmental signals locally to play a role in rapid regulation.

In conclusion, we found a mechanism by which GIT1 regulated axon growth in an inflammatory environment. This further clarified the protective effect of GIT1 on axons after SCI. In the inflammatory environment, GIT1 promoted the expression of MAP1B by regulating the ERK-Nrf2 signaling pathway. This study provided a theoretical basis for the regulation and possible treatment of spinal cord regeneration. However, the in vitro experiment in this study only simulated the growth of axons in the inflammatory state and could not simulate the state of axon injury after SCI accurately. In future studies, we plan to simulate axon damage through microfluidic devices and further explore axon regeneration.

## Data Availability

The data used to support the findings of this study are available from the corresponding authors upon request.

## Abbreviations

KO: Knockout
WT: Wild-type
GIT1: GPCR kinase 2-interacting protein-1
CSPG: Chondroitin sulfate proteoglycans
BDNF: Brain-derived neurotrophic factor
GDNF: Glial cell line-derived neurotrophic factor
ELISA: Enzyme-linked immunosorbent assay
IL-1*β*: Interleukin-1*β*
TNF-*α*: Tumor necrosis factor-*α*
MOI: Multiplicity of infection
shRNA: Short hairpin RNA
Nrf2: Nuclear factor-erythroid 2-related factor 2
PD: Parkinson's disease
mRBP: mRNA-binding protein
ZBP-1: Zipcode binding protein 1.
